# Are clinical practice guidelines for low back pain interventions of high quality and updated? A systematic review using the AGREE II instrument

**DOI:** 10.1186/s12913-020-05827-w

**Published:** 2020-10-22

**Authors:** G. Castellini, V. Iannicelli, M. Briguglio, D. Corbetta, L. M. Sconfienza, G. Banfi, S. Gianola

**Affiliations:** 1 Unit of Clinical Epidemiology, IRCCS Istituto Ortopedico Galeazzi, Milan, Italy; 2 Vita-Salute San Raffaele University, Milan, Italy; 3 IRCCS Istituto Ortopedico Galeazzi, Milan, Italy; 4 Rehabilitation and Functional Recovery Department, IRCCS Ospedale San Raffaele, Milan, Italy; 5 Department of Biomedical Sciences for Health, University of Milan, Milan, Italy

**Keywords:** Low Back pain, Spine, Clinical practice guidelines, Systematic review, Critical appraisal, AGREE II, GRADE, Quality of evidence

## Abstract

**Background:**

Clinical practice guidelines (CPGs) provide recommendations for practice, but the proliferation of CPGs issued by multiple organisations in recent years has raised concern about their quality. The aim of this study was to systematically appraise CPGs quality for low back pain (LBP) interventions and to explore inter-rater reliability (IRR) between quality appraisers. The time between systematic review search and publication of CPGs was recorded.

**Methods:**

Electronic databases (PubMed, Embase, PEDro, TRIP), guideline organisation databases, websites, and grey literature were searched from January 2016 to January 2020 to identify GPCs on rehabilitative, pharmacological or surgical intervention for LBP management. Four independent reviewers used the Appraisal of Guidelines for Research and Evaluation II (AGREE II) tool to evaluate CPGs quality and record the year the CPGs were published and the year the search strategies were conducted.

**Results:**

A total of 21 CPGs met the inclusion criteria and were appraised. Seven (33%) were broad in scope and involved surgery, rehabilitation or pharmacological intervention. The score for each AGREE II item was: Editorial Independence (median 67%, interquartile range [IQR] 31–84%), Scope and Purpose (median 64%, IQR 22–83%), Rigour of Development (median 50%, IQR 21–72%), Clarity and Presentation (median 50%, IQR 28–79%), Stakeholder Involvement (median 36%, IQR 10–74%), and Applicability (median 11%, IQR 0–46%). The IRR between the assessors was nearly perfect (interclass correlation 0.90; 95% confidence interval 0.88–0.91). The median time span was 2 years (range, 1–4), however, 38% of the CPGs did not report the coverage dates for systematic searches.

**Conclusions:**

We found methodological limitations that affect CPGs quality. In our opinion, a universal database is needed in which guidelines can be registered and recommendations dynamically developed through a living systematic reviews approach to ensure that guidelines are based on updated evidence.

**Level of evidence:**

1

**Trial registration:**

REGISTRATION PROSPERO DETAILS: CRD42019127619.

**Supplementary information:**

**Supplementary information** accompanies this paper at 10.1186/s12913-020-05827-w.

## Background

The worldwide point prevalence of Low Back Pain (LBP) is 9.4% (95% CI, 9.0–9.8) in 2010 [1]. Next to the common cold, it is one of the commonest reasons why people seek their physician, with a substantial medical social and economic impact for individuals, families, and society due to its high direct and indirect costs [2–4]. Back pain is a leading cause of years lived with disability and the first cause of activity limitation and absence from work [1]. The overall burden of LBP arising from ergonomic exposures at work was estimated at 21.8 million [95% Confidence Interval (CI) 14.5–30.5] disability adjusted life years (DALYs) in 2010 [5]. In response to the global burden, numerous CPGs have been issued by medical societies and working groups, providing recommendations for its diagnosis and management [6, 7]. While the principles for developing CPGs are well established, their proliferation has raised concern about quality. Published CPGs appraisals report that the quality is generally poor, though it appears to have recently improved, and that their applicability is generally low [8, 9]. Appraisals of CPGs for LBP [9–14] do not take into account the most recently published guidelines. Since CPGs provide a bridge between scientific literature and clinical decision making, their implementation in clinical practice should be based on recent evidence, and consider as much as possible a wide range of therapeutic choices [15].

But because 1 out of 5 recommendations in clinical guidelines go out of date within 3 years, the validity of recommendations beyond 3 years is potentially questionable [16]. As a general rule, CPGs should be reviewed every 3 years after their issue [17]. The National Institute of Clinical Excellence (NICE), the benchmark in guidelines production, has stated that “A formal review of the need to update a guideline is usually undertaken by NICE 3 years after its publication” [18]. This is warranted by the time span between the year of running the systematic search strategy during guideline production and the year of publication in a systematic review [19]. This time span is further stretched because guidelines production and dissemination need to be based on systematic reviews. The use of guidelines older than 3 years would be considered unethical in clinical decision making and mistaken in identifying high quality guidelines with not the most recent-update, available and reliable evidence [16, 17, 20].

Moreover, existing appraisals of guidelines for LBP do not rely on a comprehensive search of the many possible therapeutic options (rehabilitative, pharmacological or surgical) for treating acute and chronic LBP [21]. The scope is an important item in the AGREE II favoring guidelines that are broad in scope rather than those focusing on a particular set of interventions for a specific condition [22].

With this study, we critically appraised only the most recent evidence-based CPGs for LBP interventions by means of the AGREE (Appraisal of Guidelines Research and Evaluation) II instrument, the gold standard for critical appraisal of guidelines [22, 23], consistent with the assumption that time can influence CPG reliability. Also, we evaluated the inter-rater reliability of AGREE II and recorded the time span as the years between the date of last search and period covered by the search and guideline publication date.

## Methods

The reporting of this systematic review fulfils the Preferred Reporting Items for Systematic Reviews and Meta-Analyses [24, 25]. No ethics committee approval was needed. The protocol is registered in PROSPERO (CRD42019127619).

### Inclusion and exclusion criteria

In line with the World Health Organization, we defined a CPG as a document containing “systematically developed evidence-based statements that assist providers, patients, policy makers and other stakeholders to make informed decisions on health care and public health policy” [26].

Inclusion criteria were: (i) the systematic process evaluated the recommendations; (ii) the CPG was focused on rehabilitation, pharmacological or surgical therapeutic intervention for LBP management; (iii) the full text was published in the last 4 years (2016–2020). We used the most up-to-date version and its supplementary documents. No language restrictions were applied. Exclusion criteria were: (i) not primarily focused on LBP, such as national/international guidelines in which LBP was briefly mentioned in the context of a more comprehensive disease evaluation; (ii) not issued by a national or international society (e.g., designed for local use); (iii) declaration of recommendations was based exclusively on consensus statements or systematic reviews or commentary editorials related to published CPGs; (iv) focus on interventions other than therapeutic (e.g., prevention, diagnosis); (v) based on population subgroups (e.g., pregnant women), specific causes (e.g. spondyloarthritis) or mixed/generic population (e.g., musculoskeletal chronic pain).

### Information sources and search strategy

We systematically searched the PubMed, Embase, PEDro, and TRIP databases using the adapted terms and keywords derived from the scoping search outlined in the search strategy. We checked guideline organisation databases (e.g., National Institute for Clinical Excellence) and guideline websites (e.g., eGuidelines). Supplementary Digital Content 1 illustrates the search strategy. Two reviewers (SG, GC) with a solid background in clinical epidemiology ran the search strategy in March 2019 and updated the results in January 2020. Grey literature was searched using Google Scholar and reference lists were screened for further eligible CPGs.

### Selection of clinical practice guidelines

Search results were uploaded to Endnote software and duplicates were removed [27, 28]. Two independent reviewers (SG, VI) screened the titles and abstracts according to the eligibility criteria. Full texts were retrieved when abstracts gave insufficient information or in case of disagreement between the two reviewers. When disagreement persisted, a third reviewer was consulted (GC). Rayyan software (https://rayyan.qcri.org/) was used to manage screening and selection [29]. Reasons for study exclusion are reported.

### Appraisal of clinical practice guidelines

Four independent researchers (MB, GC, SG, VI) appraised each CPG using the AGREE II instrument and recorded with a self-chronometer the time taken for each assessment. The researchers received training in the use of AGREE II. They completed the AGREE II Online Training Tool (http://www.agreetrust.org/resource-centre/agree-ii-training-tools/) and participated in two calibration rounds with a sample of four relevant CPGs of varying quality from a previous overview of clinical guidelines for chronic LBP restricted to 2012 [30]. The original AGREE tool was published in 2003 has since then been revised in an updated version. The AGREE II instrument [22] consists of 23 items organized into six quality domains: scope and purpose, stakeholder involvement, rigour of development, clarity of presentation, applicability, and editorial independence. Supplementary Digital Content 2 shown the items and domains of the AGREE II instrument [31]. Answers to items are graded on a 7-point scale from 1 (strongly disagree) to 7 (strongly agree). A standardized score (range, 0 to 100%) was calculated for each domain.

The appraisers completed the first global rating item on a 7-point scale (1 = lowest possible quality, 7 = highest possible quality) and the second global rating item of recommending the guidelines for use in practice, with one of three options (Yes, Yes, with modifications, and No). One author (VI) calculated the standardised domain score for each of the six domains as recommended by AGREE II [22, 32]. The general data from each CPG were collected: i) authors and year of publication; ii) ex novo, update or adoption/adolopment CPG status; iii) continent of origin; iv) organization/society/association, funding source, conflict of interest. We also extracted content information such as target population, target interventions (i.e., surgery, physical therapy, pharmaceutics, educational / behavioural, alternative medicine), rating methods for the quality of evidence (e.g., the Grading of Recommendations Assessment, Development and Evaluation - GRADE), presence of a multidisciplinary panel (as defined by AGREE II: potential candidates for a panel group include clinicians, content experts, researchers, policy makers, clinical administrators, and funders; at least one methodology expert), and patient involvement (as defined by AGREE II: to capture patient/public views and preferences). Supplementary Digital Content 2.

### Data synthesis

We used descriptive statistics to summarize the characteristics of CPGs deemed eligible for inclusion. Data are summarized as frequency number (percentage) or median and interquartile range (IQR). We calculated a quality score for each of the six domains of CPGs using the formula presented in the AGREE II User’s Manual [32]. The appraisers added notes and completed the two global rating items at the end of each AGREE II assessment. The first global rating item asks appraisers to rate the overall quality of the guideline on a 7-point scale (1 = lowest possible quality and 7 = highest possible quality). Domain scores are calculated by summing up the appraisers’ scores of the individual items in a domain and then scaling the total as a percentage of the maximum possible score for that domain, which is then automatically generated on the platform My AGREE PLUS [33].

The second global rating item asks whether the appraiser would recommend the guideline for use in practice and to respond with one of three options (Yes, Yes, with modifications, and No).

The first global rating was adopted to formulate the agreement on the overall assessment between the four appraisers measuring the intraclass correlation coefficient (ICC) with 95% confidence interval (CI). The degree of agreement was graded according to Landis and Koch [34]: slight (0.01–0.2); fair (0.21–0.4); moderate (0.41–0.6); substantial (0.61–0.8); and almost perfect (0.81–1). Statistical significance was a *P* value < 0.05. All tests were two-sided [34]. All data analyses were performed using STATA (StataCorp. 2017. Stata Statistical Software: Release 15. College Station, TX, USA: StataCorp LLC).

## Results

### Search results

**Fig. 1:**
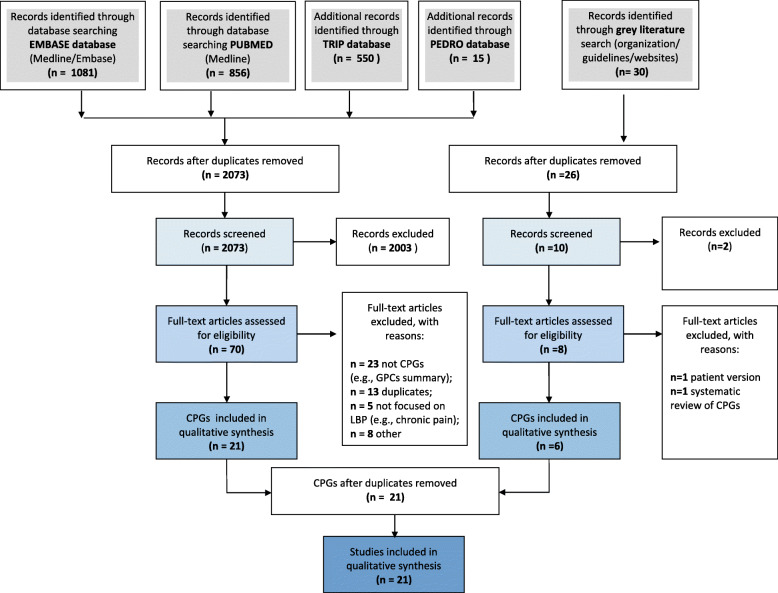
Flow diagram of CPG selection

The systematic search retrieved 2502 citations; additional 30 citations were retrieved from the grey literature. A total of 70 CPGs and related documents underwent full-text screening, 25 of which met the inclusion criteria. Four are awaiting assessment (Fig. 1). Finally, we appraised 21 CPGs using AGREE II (Supplementary Digital Content 1 and 3).

### Characteristics of CPGs

Table 1 presents the main characteristics of the 21 CPGs: 10 (47.6%) addressed multiple interventions. Rating of evidence quality was planned in 76% of the guidelines and reported in 67%. More than half (52%) had a multidisciplinary panel and less than half (38%) reported patient involvement (Supplementary Digital Content 3).

**Table 1:** Characteristics of CPGs

Clinical Practice Guideline	Publication year	Country	Status	Topic	Publication dates of systematic search strategy
**American College of Physician (ACP)** [35]	2017	USA	Update	Educational / behavioural, physical therapy, pharmaceutics	2008–2015
**American Imaging Management (AIM) Specialty Health** [36]	2019	USA	Update	Surgery	Not reported
**American Osteopathic Association (AOA)** [37]	2016	USA	Update	Physical therapy	2003–2014
**American Society of Interventional Pain Physicians (ASIPP)** [38]	2019	USA	New	Pharmaceutics	Not reported
**Brazilian Medical Association (BMA)** [39]	2018	Brazil	New	Educational / behavioural	Not reported
**China Association of Acupuncture-Moxibustion (CAAM)** [40]	2016	China	New	Alternative medicine	Not reported
**Canadian Chiropractic Guideline Initiative (CCGI)** [41]	2018	Canada	New	Educational / behavioural, physical therapy	2015–2017
**Council on Chiropractic Guidelines and Practice Parameters (CCGPP)** [42]	2016	USA	Adoption^a^	Physical therapy	2009–2014
**Change Pain Latin America (CPLA)** [43]	2018	Latin America	Adoption^b^	Physical therapy, pharmaceutics	2004–2014
**Dutch Society of Anesthesiologists (DSA)** [44]	2016	Netherlands	New	Surgery	1990–2011
**Global Spine Care Initiative (GSCI)** [45]	2018	International	New	Surgery	Not reported
**Institute for Clinical Systems Improvement (ICSI)** [46]	2018	USA	Update	Educational / behavioural, physical therapy, pharmaceutics	2000–2017
**Belgian Health Care Knowledge Centre (KCE)** [47]	2017	Belgium	Adoption^c^	Educational / behavioural, physical therapy, pharmaceutics, surgery	2010–2015
**Korea Institute of Oriental Medicine (KIOM)** [48]	2017	Korea	New	Physical therapy, pharmaceutics, alternative medicine	start date not reported - 2015
**Korean Society of Spine Surgery (KSSS)** [49]	2017	Korea	Adoption^d^	Educational / behavioural, physical therapy, pharmaceutics	2000–2016
**Labor & Industries (L&I), Washington State** [50]	2016	USA	Update	Surgery	Not reported
**National Institute for Health and Care Excellence (NICE)** [51]	2016	UK	Update	Educational / behavioural, physical therapy, pharmaceutics, surgery	2013–2015
**Polish Society of Physiotherapy (PSP)** [52]	2017	Poland	New	Physical therapy	Not reported
**Polish Spine Surgery Society (PSSS)** [53]	2016	Poland	New	Surgery	Not reported
**Toward Optimized Practice (TOP) Low Back Pain Working Group** [54].	2017	USA	Update	Educational / behavioural, physical therapy, pharmaceutics, surgery	2010–2014
**Veterans Affairs/Department of Defense (VADoD) Collaboration Office** [55]	2017	USA	Update	Educational / behavioural, physical therapy, pharmaceutics	2006–2016

^a^Three evidence-based clinical practice guidelines for the surgical and interventional management of persistent and disabling spine disorders were selected by consensus (Globe et al. J Manipulative Physiol Ther 2008; Baker et al. Top Integr Health Care, 2012; Farabaugh RJ, J Manipulative Physiol Ther 2010)

^b^Eleven evidence-based clinical practice guidelines were selected according to the field of interest. Table 4 of original publication contains all references

^c^One evidence-based clinical practice guidelines was selected (de Campos, NICE 2016);

^d^Three evidence-based clinical practice guidelines were selected according to the field of interest (Dagenais et al. Spine J 2010; Koes et al. Eur Spine J 2010; Chou et al. Ann Intern Med 200)

Note: For full details of CPG see Supplement Digital Content 3

### AGREE II domains assessment

Overall, the highest rating AGREE II domain was Editorial Independence (median 67%, interquartile range [IQR] 31–84%), followed by Scope and Purpose (median 64%, IQR 22–83%), Rigour of Development (median 50%, IQR 21–72%), Clarity and Presentation (median 50%, IQR 28–79%), Stakeholder Involvement (median 36.1%, IQR 10–74%), and Applicability (median 11%, IQR 0–46%). In the overall guideline assessment, the median of the overall quality item was 42% (IQR 15–67%) and the most frequent recommendation regarding the use of the guideline was “No” (Table 2).

**Table 2:** Overall domain assessment of CPGs

Clinical Practice Guideline		Scope and Purpose	Stakeholder Involvement	Rigour of Development	Clarity of Presentation	Applicability	Editorial Independence	OVERALL	
								first global rating (Personal rating)	second global rating (I would recommend?)
**National Institute for Health and Care Excellence (NICE)** [51]	2016	98,61%	95,83%	90,10%	100,00%	65,63%	93,75%	95,83%	Yes
**Canadian Chiropractic Guideline Initiative (CCGI)** [41]	2018	90,28%	84,72%	88,54%	90,28%	73,96%	85,42%	87,50%	Yes
**Belgian Health Care Knowledge Centre (KCE)** [47]	2017	88,89%	77,78%	83,85%	69,44%	86,46%	95,83%	83,33%	Yes
**American College of Physicians (ACP)** [35]	2017	97,22%	75,00%	76,56%	80,56%	22,92%	93,75%	75,00%	Yes
**Veterans Affairs/Department of Defense (VADoD) Collaboration Office** [55]	2017	84,72%	90,28%	80,21%	87,50%	41,67%	60,42%	70,83%	Yes, with mod.
**Institute for Clinical Systems Improvement (ICSI)** [46]	2018	80,56%	72,22%	68,23%	86,11%	51,04%	79,17%	62,50%	Yes, with mod.
**Toward Optimized Practice (TOP) Low Back Pain Working Group** [54]	2017	69,44%	48,61%	66,15%	77,78%	71,88%	75,00%	58,33%	No
**Korea Institute of Oriental Medicine (KIOM)** [48]	2017	72,22%	40,28%	47,92%	59,72%	1,04%	83,33%	45,83%	No
**China Association of Acupuncture-Moxibustion (CAAM)** [40]	2016	63,89%	44,44%	35,94%	63,89%	0,00%	29,17%	45,83%	No
**Global Spine Care Initiative (GSCI)** [45]	2018	52,78%	11,11%	25,00%	70,83%	11,46%	75,00%	41,67%	No
**American Society of Interventional Pain Physicians (ASIPP)** [38]	2019	66,67%	25,00%	57,29%	33,33%	6,25%	81,25%	41,67%	No
**American Osteopathic Association (AOA)** [37]	2016	76,39%	36,11%	52,60%	25,00%	33,33%	58,33%	41,67%	No
**Change Pain Latin America (CPLA)** [43]	2018	26,39%	13,89%	27,60%	27,78%	23,96%	93,75%	33,33%	No
**Council on Chiropractic Guidelines and Practice Parameters (CCGPP)** [42]	2016	43,06%	33,33%	53,13%	47,22%	16,67%	66,67%	29,17%	No
**Dutch Society of Anesthesiologists (DSA)** [44]	2016	63,89%	56,94%	50,52%	50,00%	4,17%	45,83%	29,17%	No
**Brazilian Medical Association (BMA)** [39]	2018	22,22%	0,00%	34,90%	29,17%	0,00%	29,17%	20,83%	No
**Korean Society of Spine Surgery (KSSS)** [49]	2017	15,28%	2,78%	17,71%	20,83%	0,00%	29,17%	8,33%	No
**Polish Spine Surgery Society (PSSS)** [53]	2016	20,83%	2,78%	12,50%	29,17%	0,00%	62,50%	8,33%	No
**American Imaging Management (AIM) Specialty Health** [36]	2019	18,06%	8,33%	6,77%	27,78%	0,00%	0,00%	4,17%	No
**Polish Society of Physiotherapy (PSP)** [52]	2017	22,22%	0,00%	1,56%	19,44%	0,00%	33,33%	4,17%	No
**Labor & Industries (L&I), Washington State** [50]	2016	12,50%	13,89%	0,00%	29,17%	0,00%	0,00%	0,00%	No

The NICE guideline [51] had the highest quality (96%) in the area of Educational/behavioural, physical therapy, pharmaceutical interventions. The Belgian Healthcare Knowledge Centre (KCE) (83%) guideline [56] had high quality and covered the same interventions plus surgery with a short time span (1 and 2 years, respectively) for searching evidence (Supplementary Digital Content 3).

### Inter-rater reliability and time for AGREE II appraisal

Inter-rater agreement was nearly perfect (ICC 0.90; 95% CI 0.88–0.91). Guidelines appraisal took 42 min on average to complete (95% CI 35–50).

### Time to publication

Overall, 38.1% of the CPGs did not report the dates of systematic search strategy, whereas less than half (47.6%) reported a median of 2 years (IQR 1–4) from search to publication. Only half provided a search within 1 year after publication (Table 1).

## Discussion

Here we report the results of quality appraisal using AGREE II of the most recent CPGs for LBP interventions (published January 2016 to January 2020) that we retrieved by systematic search of electronic medical databases and guidelines websites. A key finding was the variability in the quality of the CPGs across all six AGREE II domains; the highest average scores (> 60%) were recorded for *Domain 6 - Editorial Independence* and the *Domain 1 - Scope and Purpose* and the lowest (< 15%) for *Domain 5 - Applicability*. The overall quality was rated low and the most frequent response for guideline recommendation was “No” (15 out of 21 CPGs).

Our findings are shared by previous appraisals of CPGs for rehabilitation [57] and other contexts [8, 58, 59] that suggest room for improvement regarding rigour of development, stakeholder involvement, and applicability [8, 58, 59]. While only half of the CPGs were noted to have acceptable rigour of development *(Domain 3 - Rigour of Development),* the variability in this domain was considerable. A low score for this domain is worrying, as it has been identified as a strong predictor of quality by the AGREE instrument [8]. Regression analysis showed a statistically significant influence of the assessment of the items in this domain on overall guideline quality [60]. The item assessing the systematic search can have great importance (i.e., “Item 7: Systematic methods were used to search for evidence”) because CPGs ought to be based on recently updated evidence. However, we found that less than half did not report the time coverage of systematic search and, when reported, it ranged from 1 to 4 years before publication. Two-thirds of the CPGs in our sample adequately planned and judged the body of the evidence linked to recommendations (e.g., GRADE). However, because the application of a system for grading the evidence (i.e., GRADE) cannot always ensure inclusion of the most updated evidence within an acceptable time span, reliability should be evaluated with caution.

The validity of each recommendation, and of the CPG, is determined by the methodological quality and the transparency of its development and by the “living evidence” on which it is based. As suggested by Garcia et al., waiting more than 3 years to review a guideline is potentially too long, in which case the recommendations may be outdated by the time of guideline publication [16]. This critical issue has been addressed by the living CPGs concept [61], which draws inspiration from the established model of living systematic reviews, where evidence is continuously updated and incorporated as soon as available in the literature through a process of continuous surveillance [62]. Accordingly, AGREE II should place importance on timing and rate CPG a high-quality score when the search is conducted within 2 years of completion of the review [63].

Less than one third of the CPGs in this sample met the AGREE II criterion for participation of patients and their advocate (*Domain 2 - Stakeholder Involvement)*. Guideline developers need to prioritize patient and stakeholder involvement starting from the early stages of CPG development. They should be actively involved as members on guideline panels and their comments and inputs included in the draft guideline [64]. Furthermore, evidence suggests that involvement of patients and stakeholders leads to the inclusion of patient-relevant topics and enhances CPG implementation [65]. Unfortunately, development and implementation are erroneously considered as separate activities [8]. In our appraisal, the poorest score was recorded for CPGs applicability (*Domain 5 - Applicability*), with results similar to other CPGs in rehabilitation [57] and other conditions [8, 12, 66–68]. CPGs can provide healthcare professionals with the necessary guidance to access the best research evidence efficiently. Nonetheless, they have little effect on changing clinical behavior.

Only half of the CPGs in our sample were rated satisfactory for adequacy of the reporting of recommendations and options for management (*Domain 4 - Clarity of Presentation)*. This may be related to the purpose of AGREE II: the current version makes no distinction between quality of reporting and quality of conduct of a CPG. Despite good reporting, the methodological conduct underlying a guideline can still be weak [69]. Quality of conduct and reporting should be judged separately, just as for all other study designs [70, 71]. In systematic reviews, for instance, PRISMA and the AMSTAR assess the quality of reporting and the quality of conduct, respectively [72].

We recorded high compliance of the CPGs with the overall aim of the guideline, the clinical question, and the target population (*Domain 1 - Scope and Purpose*). This could be explained by the focus on LBP, which is the most prevalent musculoskeletal condition for which guidelines are needed in view of the years lived with disability in most countries [73]. Lastly, we recorded high compliance of the CPGs with the reporting of sources of support *(Domain 6 - Editorial Independence)*. Given the global socioeconomic burden of LBP and the need for care, CPGs must report the presence and management of conflict of interests.

### Strengths and limitations

Our appraisal has several strengths. We performed an exhaustive search that included explicit eligibility criteria and independent duplicate assessment of eligibility. Four reviewers were involved in the appraisal, with a nearly perfect inter-rater reliability. While all appraisers were trained in the use of AGREE II, it should be acknowledged that the appraisers shared a similar background (methodology and rehabilitation), which may partially explain the high overall agreement. Indeed, our team included clinical experts and methodologists with experience in clinical epidemiology, including systematic reviews and CPGs. Even after receiving the same training however, guideline appraisers from different areas may still interpret the items and the scoring system differently [74]. Furthermore, it is possible that the appraisers, basing the assessment on their own experience, paid more attention to assessing the quality of reporting than the quality of conduct and vice versa. We analysed a reliable subset of CPGs restricted to LBP in order to ensure consistency of appraisal, while avoiding discrepancies in item judgement due to different clinical contexts (e.g., AGREE II to assess CPGs in oncology differs from orthopaedics). We focused on the most recent guideline versions in order to offer stakeholders, policy makers, clinicians, and patients the latest evidence for the effectives of interventions. However, selecting the CPGs was a challenge, since the definition of guidelines is not universally established and the meaning of consensus and that of evidence-based CPG are sometimes confused. The rigour of methods and panel of experts have to be simultaneously considered in a CPG, but the current definition does not explicate these elements.

A possible limitation of our work is linked to characteristics of the AGREE II itself. It focuses on the quality of the development of CPGs, but this is not sufficient to ensure implementation of single clinical recommendations and improvement in health outcomes [75]. While high-quality CPGs can guarantee rigour in the production of recommendations, their implementation depends largely on how health care professionals decide whether or not to implement a single recommendation in the balance between content (strength and direction of a recommendation), clinical expertise, patients’ values and resources available. The implementation of a single clinical recommendation cannot be disjointed from overall CPG quality.

### Future spin for research

At the time of its publication, a CPG can already be outdated and so will not reflect the most recent evidence. Indeed, time can influence its reliability: (a) during the conduction of systematic reviews for the production of the body of the evidence needed during CPG development; (b) between finalization of a CPG and its publication. In order to avoid waste of effort and of resources due to duplication of CPGs or CPGs outdated before their time, we urge for the creation of a universal database in which guidelines can be registered and updated along the lines of registers for RCTs (e.g., WHO or clincialtrials.gov) and systematic reviews (e.g., PROSPERO) but for CPGs. In this way, a “living and dynamic” development of recommendations can be better recognized by identifying the most recent literature [76].

## Conclusion

We found methodological limitations affecting CPG quality. Our work highlights the importance of adoption of high quality and updated CPGs to guarantee the validity of a single recommendations, notwithstanding the possibility that implementation of each single recommendation may be the result of a balanced decision between content (strength and direction of a recommendation), clinical expertise, and available resources. We call for a universal database in which guidelines can be registered and recommendations dynamically developed through a living systematic reviews approach to ensure that CPGs are based on recent evidence.

## Supplementary information

**Additional file 1 Supplementary Digital Content 1.** Literature search strategy and list of CPGs appraised with AGREE II.

**Additional file 2 Supplementary Digital Content 2.** Items and domains of the AGREE II instrument.

**Additional file 3 Supplementary Digital Content 3.** Additional Characteristics of included CPGs.

## Data Availability

All data generated or analysed during this study are included in this published article with all additional materials. Row data are stored at the following link: https://osf.io/xwbu2/?view_only=d3aa81b467874b468bd1207d96df7376

## Abbreviations

ACP: American College of Physicians
AGREE II: Appraisal of Guidelines for Research and Evaluation II
AIM: American Imaging Management Specialty Health
AMSTAR: A Measurement Tool to Assess systematic Reviews
AOA: American Osteopathic Association
ASIPP: American Society of Interventional Pain Physicians
BMA: Brazilian Medical Association
CCGI: Canadian Chiropractic Guideline Initiative
CI: Confidence Interval
CPGs: Clinical Practice Guidelines
KCE: Belgian Health Care Knowledge Centre
CPLA: Change Pain Latin America
CCGPP: Council on Chiropractic Guidelines and Practice Parameters
CAAM: China Association of Acupuncture-Moxibustion
DALYs: Disability Adjusted Life Years
DSA: Dutch Society of Anesthesiologists
GSCI: Global Spine Care Initiative
GRADE: Grading of Recommendations Assessment, Development and Evaluation
IQR: Interquartile Range
ICC: Intraclass correlation
ICSI: Institute for Clinical Systems Improvement
KIOM: Korea Institute of Oriental Medicine
KSSS: Korean Society of Spine Surgery
L&I: Labor & Industries
LBP: Low Back Pain
NICE: National Institute for Health and Care Excellence
PEDro: Physiotherapy Evidence Database
PSP: Polish Society of Physiotherapy
PSSS: Polish Spine Surgery Society
PRISMA: Preferred Reporting Intervention for Systematic Review and Meta-analysis
TOP: Toward Optimized Practice Low Back Pain Working Group
TRIP: Turning Research into Practice
VADoD: Veterans Affairs/Department of Defense Collaboration Office
WHO: World Health Organization
