# Supplementary material for: Are clinical practice guidelines for low back pain interventions of high quality and updated? A systematic review using the AGREE II instrument
Source: BMC Health Serv Res. 2020 Oct 22;20:970. doi: 10.1186/s12913-020-05827-w (PMC7583191; doi:10.1186/s12913-020-05827-w)
Supplement: Supplementary file 1 — Additional file 1 Supplementary Digital Content 1. Literature search strategy and list of CPGs appraised with AGREE II. [file 12913_2020_5827_MOESM1_ESM.docx]

**Supplement Digital Content 1.**

[**Literature search strategy** 1](#_Toc49768948)

[**List of included CPGs appraised with AGREE II** 4](#_Toc49768949)

## **Literature search strategy**

1. **Search strategy run in EMBASE**

#1,"guideline*:ti"

#2,"((practice* OR clinic*) NEAR/2 guideline*):ti,ab"

#3,"'practice guideline'/exp"

#4,"'consensus development'/exp"

#5,"'consensus':ti"

#6,"(consensus NEAR/2 statement):ti,ab"

#7,"#1 OR #2 OR #3 OR #4 OR #5 OR #6"

#8,"back NEAR/1 pain"

#9,"'low back pain'/exp"

#10,"'backache'/exp"

#11,"lumba*:ti"

#12,"(lumba* NEAR/2 pain):ti,ab"

#13,"#8 OR #9 OR #10 OR #11 OR #12"

#14,"#7 AND #13"

#15,"#14 AND ('consensus development'/de OR 'practice guideline'/de) AND (2017:py OR 2018:py OR 2019:py)"

1. **Search strategy run in PUBMED**

("guideline*"[ Title/Abstract] OR "Guideline" [PT] OR "Guidelines as Topic"[Majr] OR Consensus Development Conference [PT]) AND (back pain[Mesh] OR "back pain”[Title/Abstract] OR backache[Title/Abstract] OR lumba*[Title/Abstract] OR “back disorder”[Title/Abstract]) AND (2016[dp] OR 2017[dp] OR 2018[dp] OR 2019[dp])

1. **Search strategy run in TRIP database**

(back pain OR lumbar OR backache) from: 2016; filtering: Guidelines.

1. **Search strategy run in PEDro**

Back pain; Since 2016 (filtering: Practise guidelines)

1. **Main website and indexes searched**

- Guideline Advisory Committee (<https://www.gacguidelines.ca)>;
- Canadian Medical Association infobase of clinical practice guidelines (<https://joulecma.ca/cpg/homepage)>;
- Australian National Health and Medical Research Council clinical practice guidelines (<https://www.clinicalguidelines.gov.au/>);
- eGuidelines (https://www.guidelines.co.uk/);
- Guidelines International Network (<https://www.g-i-n.net/>);
- L'agence nationale d'accréditation et d'évaluation en santé (<https://www.has-sante.fr/portail/>);
- National Institute for Clinical Excellence – NICE (<https://www.nice.org.uk/>);
- Scottish Intercollegiate Guidelines Network – SIGN (<https://www.sign.ac.uk/>);
- Istituto Superiore Sanità - Sistema Nazionale LineeGuida – ISS-SNLG (<https://snlg.iss.it>);
- New Zealand Guidelines Group (<https://www.health.govt.nz>);
- WHO (<https://www.who.int>);
- AHRQ Agency of Healthcare Research and Quality- National Guideline Clearinghouse (NGC) (<https://www.ahrq.gov>).

## **List of included CPGs appraised with AGREE II**

| **REF** | **Guideline** | **Year** | **Citation** |
| --- | --- | --- | --- |
| ACP | *Qaseem et al.* | 2017 | Qaseem A, Wilt TJ, McLean RM, Forciea MA; Clinical Guidelines Committee of the American College of Physicians. Noninvasive Treatments for Acute, Subacute, and Chronic Low Back Pain: A Clinical Practice Guideline From the American College of Physicians. Ann Intern Med. 2017 Apr 4;166(7):514-530. doi: 10.7326/M16-2367. Epub 2017 Feb 14. PubMed PMID: 28192789. Available from: <https://www.acponline.org/acp_policy/guidelines/noninvasive_treatments_for_chronic_low_back_pain_2017.pdf> |
| AIM | *AIM Specialty Health* | 2019 | AIM Specialty Health - Musculoskeletal Program - Clinical Appropriateness Guidelines for Spine Surgery.  Available from: <https://aimspecialtyhealth.com/PDF/Guidelines/2019/May18/AIM_Guidelines_MSK_Spine-Surgery.pdf> |
| AOA | *Seffinger et al.* | 2016 | Seffinger MA, Buser B, Licciardone JC, Lipton JA, Lynch JK, Patterson MM, Snow R, Troutman ME. American Osteopathic Association Guidelines for Osteopathic Manipulative Treatment (OMT) for Patients with Low Back Pain The Journal of the American Osteopathic Association (2016) 116:8 (536-549). Available from: <https://jaoa.org/article.aspx?articleid=2093924> |
| ASIPP | *Navani et al.* | 2019 | Navani A, Manchikanti L, Albers SL, Latchaw RE, Sanapati J, Kaye AD, Atluri S, Jordan S, Gupta A, Cedeno D, Vallejo A, Fellows B, Knezevic NN, Pappolla M, Diwan S, Trescot AM, Soin A, Kaye AM, Aydin SM, Calodney AK, Candido KD, Bakshi S, Benyamin RM, Vallejo R, Watanabe A, Beall D, Stitik TP, Foye PM, Helander EM, Hirsch JA. Responsible, Safe, and Effective Use of Biologics in the Management of  Low Back Pain: American Society of Interventional Pain Physicians (ASIPP) Guidelines. Pain Physician. 2019 Jan;22(1S):S1-S74. PubMed PMID: 30717500. Available from: <https://www.painphysicianjournal.com/current/pdf?article=NjEwMQ%3D%3D&journal=118> |
| BMA | *Silvinato et al.* | 2018 | Silvinato A, Simões RS, Buzzini RF, Bernardo WM, Brazilian Medical Association. Lumbar herniated disc treatment with percutaneous hydrodiscectomy. Rev Assoc Med Bras (1992). 2018 Sep;64(9):778-782. doi: 10.1590/1806-9282.64.09.778. PubMed PMID: 30672996. Available from: <http://www.scielo.br/scielo.php?script=sci_arttext&pid=S0104-42302018000900778&lng> |
| CAMM | *Zhao et al.* | 2016 | Zhao H, Liu B, Liu Z, Xie L, Fang Y, Zhu Y, Li S, Sun Y, Han M. Clinical practice guidelines of using acupuncture for low back pain. World Journal of Acupuncture - Moxibustion (2016) 26:4 (1-13). Date of Publication: 1 Dec 2016 |
| CCGI | *Bussières et al.* | 2018 | Bussières AE, Stewart G, Al-Zoubi F, Decina P, Descarreaux M, Haskett D, Hincapié C, Pagé I, Passmore S, Srbely J, Stupar M, Weisberg J, Ornelas J. Spinal Manipulative Therapy and Other Conservative Treatments for Low Back Pain: A Guideline from the Canadian Chiropractic Guideline Initiative. J Manipulative Physiol Ther. 2018 May;41(4):265-293. doi: 10.1016/j.jmpt.2017.12.004. Epub 2018 Mar 30. Available from: <https://www.chiropractic.ca/wp-content/uploads/2018/04/Bussieres-2018-Spinal-Manipulative-Therapy-and-Other-Conservative-Treatments-for-Low-Back-Pain.pdf> |
| CCGPP | *Globe et al.* | 2016 | Globe G, Farabaugh RJ, Hawk C, Morris CE, Baker G, Whalen WM, Walters S, Kaeser M, Dehen M, Augat T. Clinical Practice Guideline: Chiropractic Care for Low Back Pain. J Manipulative Physiol Ther. 2016 Jan;39(1):1-22. doi: 10.1016/j.jmpt.2015.10.006. PubMed PMID: 26804581.  Available from: <https://www.jmptonline.org/article/S0161-4754(15)00184-0/fulltext> |
| CPLA | *Amescua-Garcia et al.* | 2018 | Amescua-Garcia C, Colimon F, Guerrero C, Jreige Iskandar A, Berenguel Cook M, Bonilla P, Campos Kraychete D, Delgado Barrera W, Alberto Flores Cantisani J, Hernandez-Castro JJ, Lara-Solares A, Perez Hernandez C, Rico MA, Del Rocio Guillen Nunez M, Sempertegui Gallegos M, Garcia JBS. Most Relevant Neuropathic Pain Treatment and Chronic Low Back Pain Management Guidelines: A Change Pain Latin America Advisory Panel Consensus. Pain Med. 2018 Mar 1;19(3):460-470. doi: 10.1093/pm/pnx198. PubMed PMID: 29025132. Available from: <https://academic.oup.com/painmedicine/article/19/3/460/4093798> |
| DSA | *Itz et al.* | 2016 | Itz CJ, Willems PC, Zeilstra DJ, Huygen FJ; Dutch Society of Anesthesiologists; Dutch Orthopedic Association; Dutch Neurosurgical Society. Dutch Multidisciplinary Guideline for Invasive Treatment of Pain Syndromes of the Lumbosacral Spine. Pain Pract. 2016 Jan;16(1):90-110. doi: 10.1111/papr.12318. Epub 2015 Jun 1. Review. PubMed PMID: 26032119. |
| GSCI | *Acaroglu et al.* | 2018 | Acaroğlu E, Nordin M, Randhawa K, Chou R, Côté P, Mmopelwa T, Haldeman S. The Global Spine Care Initiative: a summary of guidelines on invasive interventions for the management of persistent and disabling spinal pain in low- and middle-income communities. Eur Spine J. 2018 Sep;27(Suppl 6):870-878. doi:10.1007/s00586-017-5392-0. Epub 2018 Jan 10. Review. PubMed PMID: 29322309. |
| ICSI | *Thorson et al.* | 2018 | Thorson D, Campbell R, Massey M, Mueller B, McCathie B, Richards H, Peterson S, Kramer C, Ginkel T, Dvorkin J, Hadzic S, Hansen A, on behalf of ICSI [Institute for Clinical Systems Improvement]. Low Back Pain, Adult Acute and Subacute. Revision date: March 2018 / 16th edition. Available from: <https://www.icsi.org/wp-content/uploads/2019/01/March-2018-LBP-Interactive.pdf> |
| KCE | *Van Wambeke et al.* | 2017 | Van Wambeke P, Desomer A, Ailliet L, Berquin A, Demoulin C, Depreitere B, Dewachter J, Dolphens M, Forget P, Fraselle V, Hans G, Hoste D, Mahieu G, Michielsen J, Nielens H, Orban T, Parlevliet T, Simons E, Tobbackx Y, Van Schaeybroeck P, Van Zundert J, Vanderstraeten J, Vlaeyen J, Jonckheer P. Low back pain and radicular pain: assessment and management. Good Clinical Practice (GCP) Brussels: Belgian Health Care Knowledge Centre (KCE). 2017. KCE Reports 287. D/2017/10.273/36. Available from: <https://kce.fgov.be/sites/default/files/atoms/files/KCE_287_Low_back_pain_Report_2.pdf> |
| KIOM | *Jun et al.* | 2017 | Jun JH, Cha Y, Lee JA, Choi J, Choi T-Y, Park W, Chung W, Shin B-C, Lee MS. Korean medicine clinical practice guideline for lumbar herniated intervertebral disc in adults: An evidence based approach European Journal of Integrative Medicine (2017) 9 (18-26). Date of Publication: 1 Jan 2017 |
| KSSS | *Hong et al.* | 2017 | Hong JY, Song KS, Cho JH, Lee JH. An Updated Overview of Low Back Pain Management in Primary Care. Asian Spine J. 2017 Aug;11(4):653-660. doi: 10.4184/asj.2017.11.4.653. Epub 2017 Aug 7. Review. PubMed PMID: 28874985.  Available from: <https://www.asianspinejournal.org/journal/view.php?doi=10.4184/asj.2017.11.4.653> |
| L&I | *L&I* | 2016 | Surgical Guideline for Lumbar Fusion (Arthrodesis) - Washington State Dept. of Labor & Industries (L&I). Available from: <https://www.lni.wa.gov/ClaimsIns/Files/OMD/MedTreat/LumbarfusionUpdate020216.pdf> |
| NICE | *De Campos et al.* | 2016 | de Campos TF. Low back pain and sciatica in over 16s: assessment and management NICE Guideline [NG59]. Available from: <https://www.nice.org.uk/guidance/ng59> |
| PSP | *Kassolik et al.* | 2017 | Kassolik k, Rajkowska-labon E, Tomasik T, Pisula-lewadowska A, Gieremek K, Andrzejewski W, Dobrzycka A. Recommendations of the Polish society of Physiotherapy, the Polish society of Family medicine and the college of Family Physicians in Poland in the field of physiotherapy of back pain syndromes in primary health care. FamMedPrimCare Rev 2017; 19(3): 323–334. doi: https://doi.org/10.5114/fmpcr.2017.69299.  Available from: <https://www.termedia.pl/Recommendations-of-the-Polish-Society-of-Physiotherapy-the-Polish-Society-of-Family-Medicine-and-the-College-of-Family-Physicians-in-Poland-in-the-field-of-physiotherapy-r-nof-back-pain-syndromes-in-p,95,30408,1,1.html> |
| PSSS | *Latka et al.* | 2016 | Latka D, Miekisiak G, Jarmuzek P, Lachowski M, Kaczmarczyk J. Treatment of lumbar disc herniation with radiculopathy. Clinical practice guidelines endorsed by The Polish Society of Spinal Surgery. Neurol Neurochir Pol. 2016;50(2):101-8. doi: 10.1016/j.pjnns.2015.12.001. Epub 2015 Dec 17. Review. PubMed PMID: 26969566. Available from: <http://ptchk.org/wp-content/uploads/2018/07/lumbar.pdf> |
| TOP | *Top* | 2017 | Toward Optimized Practice (TOP) Low Back Pain Working Group. 2017 December. Evidence-informed primary care management of low back pain: Clinical practice guideline. 3rd Edition – minor revision 2017.  Available from: <http://www.topalbertadoctors.org/cpgs/885801> |
| VADoD | *Pangarkar et al.* | 2017 | Pangarkar S, Low Back Pain Work Group. VA/DoD clinical practice guideline for diagnosis and treatment of Low Back Pain. September 2017.  Available from: <https://www.healthquality.va.gov/guidelines/Pain/lbp/VADoDLBPCPG092917.pdf> |
