# Supplementary material for: Are clinical practice guidelines for low back pain interventions of high quality and updated? A systematic review using the AGREE II instrument
Source: BMC Health Serv Res. 2020 Oct 22;20:970. doi: 10.1186/s12913-020-05827-w (PMC7583191; doi:10.1186/s12913-020-05827-w)
Supplement: Supplementary file 2 — Additional file 2 Supplementary Digital Content 2. Items and domains of the AGREE II instrument. [file 12913_2020_5827_MOESM2_ESM.docx]

|  | **Items and domains of the AGREE II instrument** |  |
| --- | --- | --- |
| **Item** | **Content** | **Domain** |
| 1 | The overall objective(s) of the guideline is (are) specifically described. | Scope and Purpose |
| 2 | The health question(s) covered by the guideline is (are) specifically described. |  |
| 3 | The population (patients, public, etc.) to whom the guideline is meant to apply is specifically described. |  |
| 4 | The guideline development group includes individuals from all relevant professional groups. | Stakeholder Involvement |
| 5 | The views and preferences of the target population (patients, public, etc.) have been sought. |  |
| 6 | The target users of the guideline are clearly defined. |  |
| 7 | Systematic methods were used to search for evidence. | Rigour of Development |
| 8 | The criteria for selecting the evidence are clearly described. |  |
| 9 | The strengths and limitations of the body of evidence are clearly described. |  |
| 10 | The methods for formulating the recommendations are clearly described. |  |
| 11 | The health benefits, side effects, and risks have been considered in formulating the recommendations. |  |
| 12 | There is an explicit link between the recommendations and the supporting evidence. |  |
| 13 | The guideline has been externally reviewed by experts prior to its publication. |  |
| 14 | A procedure for updating the guideline is provided. |  |
| 15 | The recommendations are specific and unambiguous. | Clarity of Presentation |
| 16 | The different options for management of the condition or health issue are clearly presented. |  |
| 17 | Key recommendations are easily identifiable. |  |
| 18 | The guideline describes facilitators and barriers to its application. | Applicability |
| 19 | The guideline provides advice and/or tools on how the recommendations can be put into practice. |  |
| 20 | The potential resource implications of applying the recommendations have been considered. |  |
| 21 | The guideline presents monitoring and/or auditing criteria. |  |
| 22 | The views of the funding body have not influenced the content of the guideline. | Editorial Independence |
| 23 | Competing interests of guideline development group members have been recorded and addressed. |  |

**Supplementary Digital Content 2.**
